# CO² filtration during pneumoperitoneum inflation and deflation in patients undergoing laparoscopy during the COVID-19 pandemic

**DOI:** 10.1590/0100-6991e-20202632

**Published:** 2021-01-18

**Authors:** GUSTAVO MUNAYER ABRAS, THIAGO AUGUSTUS BLASCO E SILVA, LUIZ FELIPE PIMENTA NOGUEIRA DE SOUZA LIMA, MAURO VIDIGAL DE REZENDE LOPES

**Affiliations:** 1 - Hospital Madre Teresa, Coordenador da equipe de cirurgia geral - Belo Horizonte - MG - Brasil; 2 - Faculdade de Ciências Médicas de Minas Gerais, Departamento de cirurgia - Belo Horizonte - MG - Brasil; 3 - Centro Universitário de Belo Horizonte, Departamento de cirurgia - Belo Horizonte - MG - Brasil; 4 - Hospital Madre Teresa, Médico assistente da equipe de cirurgia geral - Belo Horizonte - MG - Brasil; 5 - Hospital Madre Teresa, Especializando da equipe de cirurgia geral - Belo Horizonte - MG - Brasil; 6 - Hospital Madre Teresa, Médico assistente da equipe de pneumologia - Belo Horizonte - MG - Brasil

**Keywords:** Coronavirus Infections, Surgery, Virology, Respiration, Artificial, Infecções por Coronavírus, Cirurgia, Virologia, Respiração Artificial

## Abstract

The current Covid-19 pandemic has been the most discussed topic of the year, mostly about protection and ways to avoid dissemination of the virus. In the healthcare system, especially in the operating rooms, the viability of laparoscopic surgery was questioned, mostly because of the transmission through aerosol. This article tries to suggest a way to minimize risks of laparoscopic surgery, during this situation, by using electrostatic filters, a simple, effective and low cost alternative.

## RESUMO

A atual pandemia do Covid-19 tem sido o assunto mais discutido do ano de 2020, principalmente no que se refere a proteção e as formas de limitar a disseminação do vírus. No cenário hospitalar, mais especificamente no centro cirúrgico, a viabilidade da cirurgia laparoscópica foi questionada, em relação a transmissibilidade do vírus por aerossol. Este artigo sugere uma forma de minimizar os riscos em cirurgias laparoscópicas durante esse cenário, com o uso de filtros eletrostáticos de ventilação mecânica. Uma alternativa simples, eficaz e de baixo custo.

COVID-19 is certainly the most discussed topic both by the scientific community and the lay media in the first semester of 2020^1^. Among the many discussed topics, the potential transmission pathways and the adopted measures to avoid its dissemination dominate the discussions^2^. 

Once the virus might be transmitted by aerosol, the viability of the laparoscopic procedures has been questioned, once it is known that other virus, as the B hepatitis, maybe in the environment air while insufflating and emptying the pneumoperitoneum^3^. The latter has led many medical societies to contraindicate the minimum invasive operations in the last months^4^. 

Bearing in mind the decreased morbidity and all the other advantages related to videolaparoscopy when compared to the conventional surgical approach, we have implemented safety protocols that allow us to carry out these procedures, guaranteeing patient safety as well as of those in the surgical room. 

The electrostatic hygroscopic filters used for mechanical ventilation are of low cost and are easily available in the surgical room. This type of filter has an efficiency of 99.99% against bacteria and virus, such as those of B and C hepatitis; they have a diameter of 42nm and 30-60nm, respectively^5^. The SARS-COV-2 has a bigger diameter, approximately 80nm, therefore, the same efficacy could be feasible. However, this requires further scientific confirmation^3^. 

The system is put together in a very easy and simple manner, by the interposition of the electrostatic filter and an intravenous line between the insufflation/draining system and the trocar, as shown in the below pictures. 

**Figure 1:**
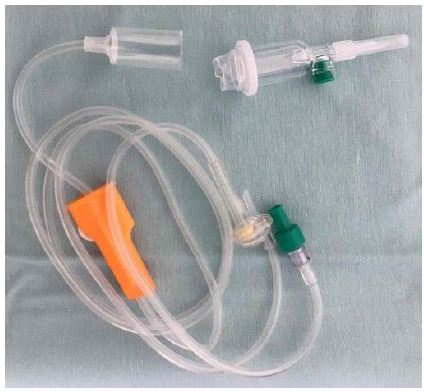
Cutting of the line chamber.

Figure 1. Cutting of the line chamber. 

**Figure 2:**
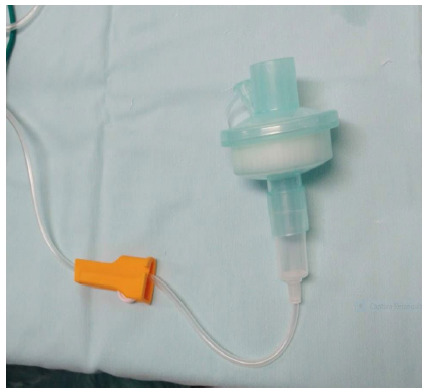
The line chambre fits into the electrostatic filter.

Figure 2. The line chambre fits into the electrostatic filter. 

**Figure 3:**
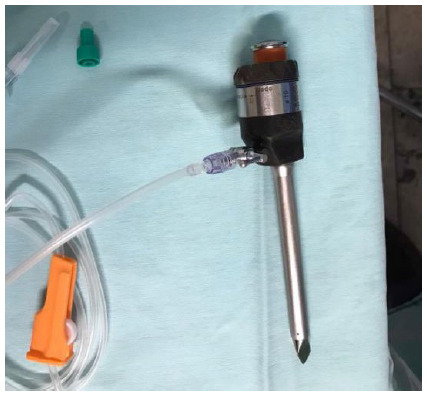
The other line extremity is connected to the trocar.

Figure 3. The other line extremity is connected to the trocar.

**Figure 4:**
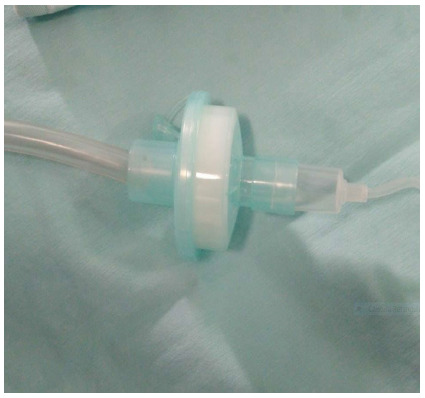
The line chambre fits into the electrostatic filter.

Figure 4. The electrostatic filter connects the insufflator tube and the line. 

There could be aerosol formation beyond the process of insufflation and drainage of the pneumoperitoneum^6^. Therefore, we strongly recommend that not only strict care should be taken for the CO^2^ drainage, but also several other safety processes should be adopted during the COVID-19 pandemic^1^. 

The use of personal protective equipment, the decrease of the number of circulating personnel in the surgical room, in particular during intubation and extubation, as well as a low intraperitoneal pressure throughout the operation are among the extra care that should be taken^6^.

Considering that appendectomy is the most common urgent procedure carried out by videolaparoscopy, we recommend the videoassisted technique whose effectivity and safety has been well demonstrated^7^. Under this technique there is less pneumoperitoneum exposition time. Furthermore, there is no need for the use of the electrocautery which could also be responsible for aerosol formation^8^. 

The CO^2^ filtration during the pneumoperitoneum insufflation and drainage, along all the other measures, has added extra care to guarantee safety during the pandemic. These are easy and low cost attitudes that minimize the risks of videolaparoscopy.
